# Selection and Evaluation of Reference Genes for Expression Analysis Using qRT-PCR in the Beet Armyworm *Spodoptera exigua* (Hübner) (Lepidoptera: Noctuidae)

**DOI:** 10.1371/journal.pone.0084730

**Published:** 2014-01-15

**Authors:** Xun Zhu, Miao Yuan, Muhammad Shakeel, Youjun Zhang, Shaoli Wang, Xin Wang, Sha Zhan, Tinghao Kang, Jianhong Li

**Affiliations:** 1 Laboratory of Pesticide, College of Plant Science & Technology, Huazhong Agricultural University, Wuhan, China; 2 Department of Plant Protection, Institute of Vegetables and Flowers, Chinese Academy of Agricultural Sciences, Beijing, China; Ghent University, Belgium

## Abstract

Quantitative real-time PCR (qRT-PCR) is a reliable and reproducible technique for measuring and evaluating changes in gene expression. The most common method for analyzing qRT-PCR data is to normalize mRNA levels of target genes to internal reference genes. Evaluating and selecting stable reference genes on a case-by-case basis is critical. The present study aimed to facilitate gene expression studies by identifying the most suitable reference genes for normalization of mRNA expression in qRT-PCR analysis of the beet armyworm *Spodoptera exigua* (Lepidoptera: Noctuidae). For this purpose, three software tools (geNorm, NormFinder and BestKeeper) were used to investigate 10 candidate reference genes in nine developmental stages and five different tissues (epidermis, head, midgut, fat body and hemolymph) in three larval physiological stages (molting, feeding and wandering stages) of, *S. exigua*. With the exception of 18S ribosomal RNA (18S), all other candidate genes evaluated, β-actin1(*ACT1*), β-actin2 (*ACT2*), elongation factor1(*EF1*), elongation factor 2 (*EF2*), Glyceralde hyde-3-phosphate dehydrogenase (*GAPDH*), ribosomal protein L10 (*L10*), ribosomal protein L17A (*L17A*), superoxide dismutase (*SOD*), α-tubulin (*TUB*),proved to be acceptable reference genes. However, their suitability partly differed between physiological stages and different tissues. *L10, EF2* and *L17A* ranked highest in all tissue sample sets. *SOD, ACT2, GAPDH, EF1* and *ACT1* were stably expressed in all developmental stage sample sets; *ACT2, ACT1* and *L10* for larvae sample sets; *GAPDH, ACT1* and *ACT2* for pupae and adults; *SOD* and *L17A* for males; and *EF2* and *SOD* for females. The expression stability of genes varied in different conditions. The findings provided here demonstrated, with a few exceptions, the suitability of most of the 10 reference genes tested in tissues and life developmental stages. Overall, this study emphasizes the importance of validating reference genes for qRT-PCR analysis in *S. exigua*.

## Introduction

Quantification of gene expression levels is fundamentally important for identifying genes relevant to biological processes [1] and provides insights into complex regulatory networks. Quantitative real-time PCR (qRT-PCR) [2], [3] is one of the most reliable and reproducible techniques available to measure and evaluate changes in gene expression[4], which is often used to confirm or refute interpretations of relative gene expression profiles derived from high-throughput systems [4], [5]. The qRT-PCR technique is sensitive enough to detect subtle alterations in gene expression, even for those with fairly low transcript levels [6], [7]. Although this powerful technique is often described as the gold standard, results are inevitably affected by different experimental conditions, such as different amounts of starting material, quality and integrity of template RNA samples, reverse transcription efficiency, recovery and integrity of mRNA, primer design and transcription efficiency [8]. Additionally, random pipetting errors can add technical variability to the data [9], [10]. As these factors can potentially render the quantification of gene transcripts unreliable, having a robust system for normalization of qRT-PCR data is essential to avoid non-specific variations or errors [6], [11]. The most common method for normalizing gene expression levels is to compare mRNA levels of the genes of interest to those of endogenous control genes, which are often called housekeeping or reference genes.

Ideal reference genes should not be regulated or influenced by the experimental procedure or co-regulated with the target gene. They should also be expressed in abundance and have minimal innate variability [12]. However, the indiscriminate use of some internal reference genes is questionable, since their expression levels are regulated according to cellular conditions [13]–[15]. Several studies have shown that this approach can introduce large errors when the expression of such “housekeeping genes” varies under different treatments and in different tissues [16], [17].

The beet armyworm, *Spodoptera exigua* (Lepidoptera: Noctuidae), is a widespread and polyphagous lepidopteran pest that causes severe economic damage in both dicotyledon (e.g., sugar beet, alfalfa, cotton, chrysanthemum) and monocotyledon (e.g., rice) crops and flower species. Molecular studies have been widely conducted previously in *S. exigua* [18]–[20], including investigations of insecticide resistance [21]–[23] and the role of important genes in physiological processes of the insect [24]. Understanding the function of important regulatory genes at the molecular level is essential for pest control. The molting, feeding and wandering stages are three larval physiological stages of the Lepidoptera larvae, which are regulated by different specific hormone levels. Tissues and genes in these three larval physiological stages have shown significant differences [25]–[27]. Therefore, molecular studies directed towards the three larval physiological stages are at the center of Lepidoptera physiological research. The fat body is a major tissue found to play an important role in the metabolism and detoxification of xenobiotics in insects [28], [29]. Receptors involved in insecticide resistance have been found in the midgut [30], [31], and several antiviral proteins and genes of potential value in clinical medicine were found in the epidermis and hemolymph [32]. Exploring gene expression profiles in these tissues will help our understanding of the regulation of the three larval physiological stages and facilitate application of useful resources to control the pest. Several genes have been demonstrated to be differentially expressed in some tissues based on sex [33], and some genes related to insecticide resistance, such as P450, are regulated by female mating [34]. Some studies have also reported differences in expression of the sex pheromones of *S. exigua* [35].

To date, studies have been published on evaluating the stability of reference genes in some insects [36], [37], [38]. Teng *et al.* [39] chose 4 candidates and screened the relatively stable reference genes in 4 lepidopteran insect species including *S. exigua*. Several studies have shown that each candidate reference gene should be evaluated under specific experimental conditions for gene expression profiling to ensure that expression occurs at a constant level [40]. The evaluation and selection from just four candidate genes seemed to be insufficient. Therefore, ten commonly used reference genes β-actin1(*ACT1*), β-actin2 (*ACT2*), elongation factor1(*EF1*), elongation factor 2 (*EF2*), Glyceralde hyde-3-phosphate dehydrogenase (*GAPDH*), ribosomal protein L10 (*L10*), ribosomal protein L17A (*L17A*), superoxide dismutase (*SOD*), α-tubulin (*TUB*), 18S ribosomal RNA (*18S*) from *S. exigua* were tested and their effectiveness for the normalisation of expression studies were further validated by quantitative analysis of a well-studied target diapause-specific peptide (DSP) gene. Three available and commonly used tools (geNorm, NormFinder and BestKeeper) were used to determine a set of the most stably expressed genes in different developmental stages (egg, 1^st^ larvae, 2^nd^ larvae, 3^rd^ larvae, 4^th^ larvae, 5^th^ larvae, prepupae, pupae and adult), in both sexes of pupae and adults, as well as in five different tissues (epidermis, head, midgut, fat body and hemolymph) and three larval physiological stages (molting stage, feeding stage and wandering stage) of *S. exigua*. The objectives of this investigation were (i) to provide appropriate reference genes to develop an accurate and comprehensive qRT-PCR method for use in *S. exigua* studies, and (ii) to assess the importance of variations in relative quantification among normalization strategies in different developmental stages, sexes, larval physiological stages and tissues, with a focus on the merits of using multiple versus single reference genes in different studies.

## Materials and Methods

### Insects

*S. exigua* were reared on an artificial diet [41] at 27±1°C (14L: 10D). Pupae were selected and sexed on the third day. Adult males and females were allowed to emerge in transparent containers and fed with a 5% honey solution.

### Sample collection

The stability of candidate genes was tested in different *S. exigua* samples of (i) five different tissues in three larval physiological stages, (ii) different developmental stages and (iii) two sexes. Only the tissue samples in three larval physiological stages had been dissected individually and all other samples were whole body. For each of the different sample groups, three replicate cages were used.

### Sampling of different tissues in three larval physiological stages

For this study, the beet armyworms were synchronized in the 4^th^ larval molting stage, 5^th^ larval feeding stage (48 h post-molting) or 5^th^ larval wandering stage (96 h post-molting), and then larvae in the three larval physiological stages were dissected individually using a dissection needle in physiological saline. The epidermis, head, midgut, fat body and hemolymph were collected separately. The collected tissues were quickly frozen and homogenized immediately after dissection with liquid nitrogen in a mortar and used for RNA extraction.

### Samples of different developmental stages

The beet armyworms in different developmental life stages were collected separately and pooled as follows: eggs (50–80 per pool), 1^st^ larvae (50–80 per pool), 2^nd^ larvae (50–80 per pool), 3^rd^ larvae (10 per pool), 4^th^ larvae (10 per pool), 5^th^ larvae (10 per pool), prepupae (10 per pool), pupae (10 per pool) and adults (10 per pool).

### Samples of different sexes

Male pupae (10 per pool), female pupae (10 per pool), male adults (10 per pool) and female adults (10 per pool), were collected separately and placed in 1.5 ml centrifuge tubes.

### Selection of gene sequences and primer design

PCR primer sequences used for quantification of the 10 candidate genes are shown in Table 1. The secondary structure of the DNA template was analyzed with UNAFold[42] using the mfold web server (http://mfold.rna.albany.edu/?q=mfold/DNA-Folding-Form) [43] with the following settings: melting temperature, 60°C; DNA sequence, linear, Na^+^ concentration, 50 mM; Mg^++^ concentration, 3 mM. Other parameters were set by default. The primers were designed using NCBI Primer-BLAST (http://www.ncbi.nlm.nih.gov/tools/primer-blast/index.cgi?LINK_LOC=BlastHome), with the settings: primer melting temperature, 60°C; primer GC content, 40–60%; and PCR product size, 80–200 base pairs. The excluded regions were based on results of analysis by mfold, and other parameters were set by default.

**Table 1:** Description, primer sequence and amplicon characteristics for the 10 candidate reference genes and a target gene used in this study.

Gene symbol	Gene name	(putative)Function	Gene ID	Primer sequences [5′→3′]	L(bp)^a^	E(%)^b^	*r^2^* c	slope	y intercept
*ACT1*	β-actin1	Involved in cell motility, structure and integrity	AEJ38214.1	*For 5*′ AAGCCTTCGATGCCACCGGGTA *3*′ *Rev 5*′ TTCGGGCGTGTTTAGTGGAGGC *3*′	170	107.3	0.997	−3.15857	52.363
*ACT2*	β-actin2	Involved in cell motility, structure and integrity	AEJ38216.1	*For 5*′ GGCTGCCGACATAGACATGCG *3*′ *Rev 5*′ GGGTCCTCCACGCGGATCTT *3*′	180	107	0.996	−3.16485	51.900
*EF1*	elongation factor1	Catalysation of GTP-dependent binding of amynoayl-total RNA to the ribosome	AEJ38219.1	*For 5*′ TGCAGAGAAGCAAGTATTTGAGCGA *3*′ *Rev 5*′ CCACGAGCTTTCTCTTCCGGAGC *3*′	180	101.5	0.990	−3.2865	57.719
*EF2*	elongation factor 2	Catalysation of GTP-dependent binding of amynoayl-total RNA to the ribosome	AAL83698.1	*For 5*′ CTGACCGCGCAACCCAGACT *3*′ *Rev 5*′ CACGAACATGGGGGTACCAGCG *3*′	150	99.4	0.998	−3.33639	51.986
*GAPDH*	Glyceralde hyde-3-phosphate dehydrogen ase	Glycolytic enzyme	AEJ38217.1	*For 5*′ CTGAGGAACAGGTCGTGTCATCCGA *3*′ *Rev 5*′ GATCGATAACGCGGTTGGAGTAGCC *3*′	150	98.5	0.996	−3.3584	50.509
*L10*	ribosomal protein L10	Structural constituent of ribosome	ABX54738.1	*For 5*′ GGCTACGGTCGACGACTTCCC *3*′ *Rev 5*′ GCAGCCTCATGCGGATGTGGAAC *3*′	155	102.6	0.997	−3.26116	52.455
*L17A*	ribosomal protein L7A	Structural constituent of ribosome	ABX55885.1	*For 5*′ TGAGCTTGTCCTCTTCCTGCCC *3*′ *Rev 5*′ GCTGCACGGTCGCCAGACTC *3*′	150	101	0.996	−3.2982	51.733
*SOD*	Superoxide dismutase	Highly specific superoxide dismutation activity	ABX11259.1	*For 5*′ GCCGTGTGTGTTCTCAAGGGCG *3*′ *Rev 5*′ GCGCCAGCTGACGTGCATCC *3*′	170	101.3	0.993	−3.29117	53.057
*TUB*	α-tubulin	Cytoskeleton structural protein	ADL38966.1	*For 5*′ CGTGACGACGTGTCTGCGGT *3*′ *Rev 5*′ GCGTGAGCTCGGGTACGGTG *3*′	167	100.2	0.998	−3.31714	55.528
*18S*	18S ribosomal RNA	Cytosolic small ribosomal subunit	JN863293.1	*For 5*′ GGTCCATCACGATGCGGTGGG *3*′ *Rev 5*′ TACCCAATCGCAACCGAGCAACG *3*′	150	111.4	0.991	−3.07593	55.544
*DSP*	diapause-specific peptide	An endogenous diapause -specific peptide; antifungal activity	HQ128581.1	*For 5*′ ATGGCCGCTCTCAAGACCAC *3*′ *Rev 5*′ TCATCAGTAACAGTCCATCCTACCG *3*′	195	108.3	0.995	−3.13785	58.553

^a^ Amplicon length;

^b^ Real-time qPCR efficiency (calculated by the standard curve method);

^c^ Regression coefficient calculated from the regression line of the standard curve.

### Total RNA isolation and cDNA synthesis

All collected samples were preserved in microcentrifuge tubes (1.5 ml) and stored at −80°C after being frozen in liquid nitrogen. Subsequently, three total RNA samples were prepared for each sample set using the SV Total RNA Isolation System (Promega, USA). According to the protocol of the kit, total RNA was incubated for 15 minutes at 20–25°C after adding 5 µl DNase I enzyme (Promega, USA). The purified RNA was stored at −80°C before further processing. The quality and quantity of RNA were assessed with a UV-1800 spectrophotometer (Shimadzu, Japan). cDNA was produced using the PrimeScript 1st Strand cDNA Synthesis Kit (TAKARA, Japan) in a total volume of 20 µl, with 4 µl 5×PrimeScript Buffer,1 µg of total RNA, 1 µl oligo dT primer, 1 µl PrimeScript RTase (200 U/µl), and 0.5 µl RNase Inhibitor (40 U/µl). Following the manufacturer's protocol, the 20 ul mixture was incubated for 60 minutes at 42°C. No-template and no-reverse transcription (no-RT) controls were run for each reverse transcription run for the control treatment. cDNA was stored at−20°C until used.

### qRT-PCR

Triplicate first strand DNA aliquots for each sample served as templates for qRT-PCR using SoFast™ EvaGreen® Supermix (Bio-Rad, USA) on an iQ2 Optical System (Bio-Rad). Each amplification reaction was performed in a 20 µl total volume with 1 µl of cDNA and 100 nM of each primer in an iQ™ 96-well PCR plate (Bio-Rad), which was covered with Microseal “B” adhesive seals (Bio-Rad). Thermal cycling conditions included initial denaturation at 95°C for 30 s, followed by 40 cycles of 95°C for 5 s and 60°C for 10 s. After all reactions, a melting curve analysis from 65 to 95°C was applied to ensure consistency and specificity of the amplified product. A 10-fold dilution series of cDNA from the whole body of adults was employed as a standard curve, and the qRT-PCR efficiency was determined for each gene and each treatment with the slope of a linear regression model [44]. The corresponding qRT-PCR efficiencies (E) were calculated according to the equation: E = (10^[−1/slope]^ −1)×100 [45].

### Stability of gene expression

The stability of candidate genes was evaluated by three commonly used software tools, BestKeeper [46], [47], geNorm (http://medgen.ugent.be/~jvdesomp/genorm/)[48] and NormFinder (http://www.mdl.dk/publicationsnormfinder.htm) [49]. The Excel based tool Bestkeeper, is able to compare expression levels of up to ten housekeeping genes together with ten target genes, each up to hundred biological samples. The raw data of cycle threshold (Ct) values(CP values) and PCR efficiency (E) of the candidate genes were used to determine the best-suited standards by BestKeeper. The underlying principle for identification of stably expressed reference genes by Bestkeeper is that the expression levels of suitable reference genes should be highly correlated. Therefore, the correlation between each candidate and the index is calculated, describing the relation between the index and the contributing candidate reference gene by the coefficient of determination and the P value [46].Ct values converted to linear values (the lowest relative quantity for each gene was set to 1) were used as input data for subsequent analyses with geNorm and NormFinder. Similar with Bestkeeper, the key principle of geNorm is that the expression ratio of two suitable reference genes should be constant across samples. geNorm algorithm first calculates an expression stability value (M) for each gene and then compares the pairwise variation (V) of this gene with the others. Using microarray data as a training set for the algorithm, the value of Vn/Vn+1 indicates the pairwise variation between two sequential normalization factors and determines the optimal number of reference genes required for accurate normalization. A value below 0.15 indicates that an additional reference gene will not significantly improve normalization. Reference genes are ranked according to their expression stability by a repeated process of stepwise exclusion of the least stably expressed genes [48]. NormFinder provides a stability value for each gene which is a direct measure for the estimated expression variation enabling the user to evaluate the systematic error introduced when using the gene for normalizsation [49]. Every gene was ranked by the three software tools and assigned an appropriate weight separately. The final ranking was established after calculating the geometric mean of their weights.

### Evaluation of target gene expression

*DSP* of *S. exigua* was used as a target gene to evaluate the candidate reference genes. Normalized with different reference genes, relative quantification of *DSP* in different samples were conducted according to threshold cycle (Ct) value based on 2^−△△Ct^ method.

## Results

### Amplification efficiencies

The initial screening of 10 candidate reference genes and one target gene by PCR showed that all of the genes were expressed in all *S. exigua* sample sets, as indicated by the presence of a single amplicon of the expected size on a 2% agarose gel. In order to determine the amplification efficiency of all 11 genes in the study, 5-point standard curves with known concentrations of transcribed reference RNA were made. All amplification efficiencies in the qRT-PCR analysis for the 10 candidate genes and one target gene ranged between 98.5∼111.4% compared with the templates from which the primers were designed. Linear regression coefficients (*r^2^*) for all 11 genes were ≥0.990 (Table 1).

### Expression levels of 10 candidate reference genes

**Figure 1:**
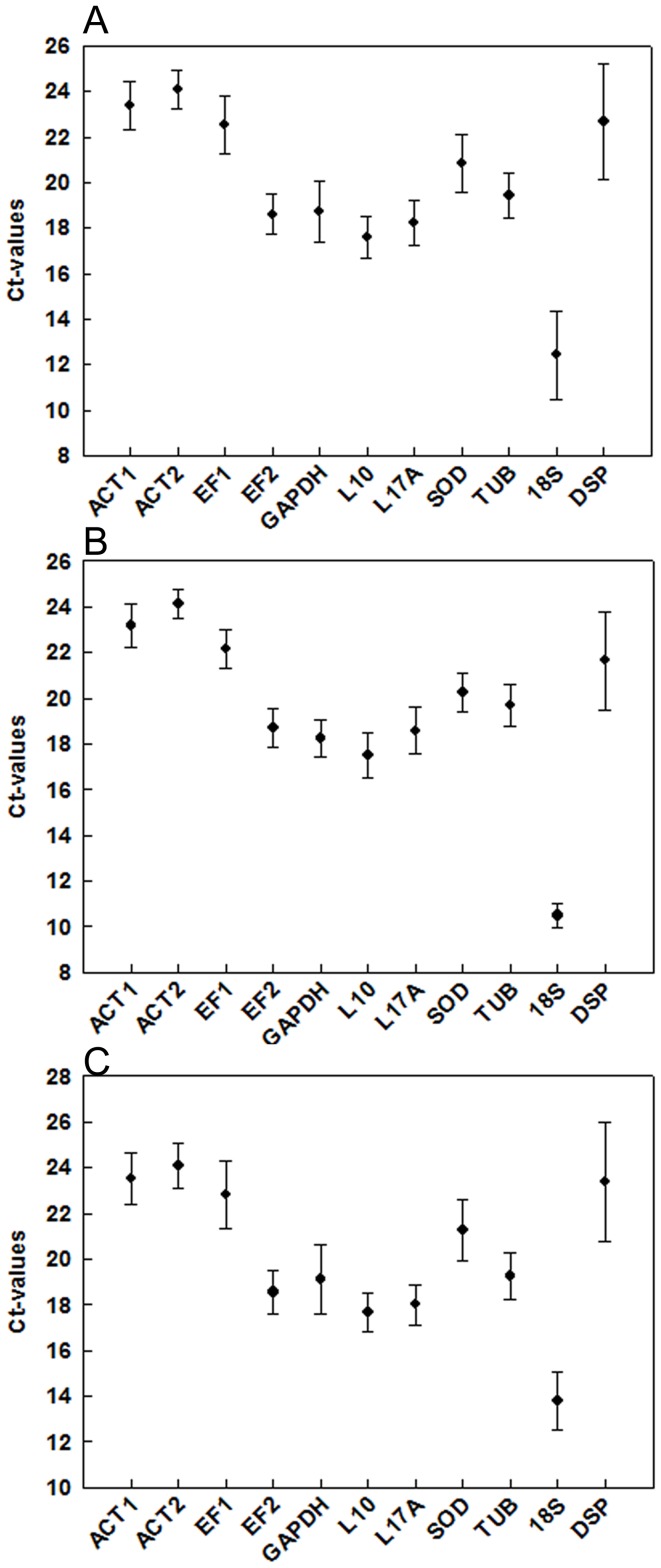
Range of Ct values in different developmental stages and tissues *of S.exigua*. The above plots show expression levels of 10 candidate reference genes and a target gene in (A) all *S. exigua* samples (n = 26), (B) different developmental stage samples (n = 11) and (C) all tissues samples in three larval physiological stages (n = 15). Values are given as Ct values from the mean of duplicate samples. Bars indicate standard error of the mean.

Relative Ct values are widely used as a simple way to identify stably expressed genes by qRT-PCR. Gene expression analyses of the 10 candidate genes exhibited a narrow mean Ct value range across all the experimental samples (Figure 1). The Ct values of the candidate reference genes under the same threshold value for fluorescence ranged from 9.18 for *18S* to 26.00 for *ACT2*, which were the most and least abundant transcripts, respectively. There were no much differences among the average Ct values for each gene, and the range of values was consistently narrower in individuals than in tissue samples, when the two sample sets consisting of the developmental stage samples and the tissue ones in three larval physiological stages were compared (Figure 1B and 1C). The amplification of *18S*, which was generally highly expressed, produced much lower Ct values (mean Ct  = 12.41) than did other genes overall. The other candidate reference genes were expressed at moderate levels, with mean Ct (n = 26 samples) values of 23.37, 24.11, 22.53, 18.56, 18.75, 17.62, 18.25, 20.85 and 19.45 for *ACT1*, *ACT2*, *EF1*, *EF2*, *GAPDH*, *L10*, *L17A*, *SOD* and *TUB*, respectively (Figure 1A). The Ct values obtained for the target gene *DSP* varied in different samples, ranging from 18.23 (female pupae) to 27.51 (midgut of 5^th^ feeding larval stage). Therefore, the standard errors of Ct values obtained for *DSP* were larger than those of all of the candidate reference genes studied (Figure 1).

### Expression stability of candidate reference genes

Ct values of the 10 candidate reference genes were obtained in each sample, and variations in their expression were assessed by Bestkeeper. Ct values converted to linear values were used as input data for subsequent analyses with geNorm and NormFinder.

### BestKeeper analysis

The high correlation of expression levels is the key principle for identification of stable reference genes, which ideally should display similar expression patterns across samples. The program BestKeeper was used to determine variations in expression and standard deviations (SD) of 10 candidate reference genes and a target gene. Examination of the standard deviations (SD) (Table 2) revealed that the candidate reference genes were not all stable across different samples, because some showed SD values higher than 1.0. The variations were diverse in different sample groups.

**Table 2:** Descriptive statistic analysis with BestKeeper.

		ACT1	ACT2	EF1	EF2	GAPDH	L10	L17A	SOD	TUB	18S	DSP
**Specific Larval Physiological Stages**												
**Molting**	SD (±CP^a^)^b^	0.982	0.9	1.132	0.678	0.996	0.681	0.689	0.978	0.462	1.09	2.451
**Stage**	BK Corr [r]^c^	0.688	0.771	0.942	0.962	0.9	0.971	0.968	0.937	0.667	0.77	−0.025
**Feeding**	SD (±CP)	0.744	0.757	1.128	0.931	1.513	0.835	0.75	1.194	0.579	1.111	2.734
**Stage**	BK Corr [r]	0.556	0.675	0.937	0.959	0.967	0.983	0.97	0.952	0.846	0.907	0.469
**Wandering**	SD (±CP)	0.864	0.643	0.987	0.497	0.885	0.528	0.692	0.608	1.009	0.665	1.136
**Stage**	BK Corr [r]	0.773	0.746	0.836	0.821	0.81	0.902	0.902	0.479	0.862	−0.083	0.257
**Five tissues in different stages**												
**Total** d	SD (±CP)	0.882	0.757	1.224	0.672	1.322	0.677	0.73	1.133	0.763	0.94	2.267
	BK Corr [r]	0.618	0.712	0.824	0.923	0.84	0.965	0.937	0.781	0.601	0.685	0.231
**Epidermis**	SD (±CP)	0.486	0.228	0.668	0.25	0.697	0.149	0.14	0.87	0.624	0.534	1.675
	BK Corr [r]	0.411	0.647	0.552	0.101	0.746	0.343	0.402	0.831	−0.023	−0.186	0.111
**Fat body**	SD (±CP)	0.531	0.507	0.855	0.358	0.938	0.312	0.412	1.186	0.528	0.466	1.534
	BK Corr [r]	0.4	0.452	0.663	0.84	0.742	0.909	0.795	0.797	−0.016	−0.05	0.302
**Head**	SD (±CP)	0.685	0.556	0.816	0.292	1.112	0.228	0.226	1.01	0.823	0.475	0.918
	BK Corr [r]	0.788	0.909	0.042	0.872	0.763	0.73	0.657	0.801	0.331	0.467	0.308
**Hemolymph**	SD (±CP)	0.858	0.779	0.886	0.838	0.688	0.686	0.571	1.386	0.864	0.888	1.584
	BK Corr [r]	0.946	0.953	0.908	0.982	0.942	0.967	0.955	0.901	0.903	0.953	0.874
**Midgut**	SD (±CP)	1.177	1.165	1.771	0.604	1.11	0.745	0.939	0.835	0.865	0.486	1.905
	BK Corr [r]	0.979	0.994	0.994	0.908	0.838	0.983	0.976	0.839	0.972	0.461	−0.798
**Developmental life stages**												
**Developmental**	SD (±CP)	0.688	0.481	0.654	0.661	0.645	0.778	0.81	0.714	0.743	0.471	1.572
**life stages** e	BK Corr [r]	0.639	0.647	0.743	0.538	0.689	0.683	0.728	0.865	0.633	0.223	−0.172
**Larvae**	SD (±CP)	0.287	0.244	0.298	0.601	0.593	0.436	0.538	0.422	0.531	0.473	1.157
	BK Corr [r]	0.573	0.423	0.761	0.849	0.809	0.919	0.808	0.463	0.714	−0.335	0.276
**Pupae**	SD (±CP)	0.432	0.348	0.442	0.251	0.2	0.26	0.331	0.468	0.551	0.576	1.092
	BK Corr [r]	0.949	0.95	0.731	0.772	0.892	0.851	0.782	0.953	0.933	0.955	−0.599
**Adult**	SD (±CP)	0.859	0.615	0.917	0.329	0.71	0.447	0.335	0.77	0.779	0.404	0.552
	BK Corr [r]	0.968	0.978	0.838	0.917	0.942	0.861	0.906	0.966	0.954	0.023	−0.163
**Sex**												
**Male**	SD (±CP)	0.369	0.369	0.751	0.378	0.847	0.931	0.539	0.46	0.515	0.432	0.553
	BK Corr [r]	0.186	0.3	0.576	−0.238	0.758	0.723	0.88	0.789	0.822	0.728	−0.161
**Female**	SD (±CP)	1.496	1.159	0.564	0.853	0.173	0.297	0.221	0.958	1.048	0.466	1.08
	BK Corr [r]	0.942	0.942	0.828	0.91	0.175	−0.55	0.087	0.971	0.951	0.574	−0.634

^a^ CP: Crossing point;

^b^ SD (±CP): the standard deviation of the CP;

^c^ BK CorrC [r]: Pearson correlation coefficient, correlation between the *BestKeeper* index and the contributing gene;

^d^ Total, all the tissues samples in three specific larval physiological stages;

^e^ Developmental Stages, all the developmental life stages samples.

Due to high variability as presented with SD (± CP) >1.0, the following genes in the indicated samples were excluded: *EF1*, *GAPDH*, and *SOD* in all tissues samples; *EF1* and *18S* in molting stage samples; *EF1*, *GAPDH*, *SOD*, and *18S* in feeding stage samples; *TUB* in wandering stage samples; *SOD* in fat body and hemolymph samples; *GAPDH* and *SOD* in head samples; *ACT1*, *ACT2*, *EF1*, and *GAPDH* in midgut samples; and *ACT1*, *ACT2*, and *TUB* in female samples. The target gene *DSP* showed the highest variations with SD values of nearly 1.0. Other genes in each experimental condition were ranked based on Pearson's correlation coefficient (a higher coefficient indicates greater stability of expression) (Table 2). Interestingly, despite displaying acceptable stability levels (i.e., far below the default limit of SD 1.0), *SOD* had the highest standard deviation, indicating that it was the least stable of the candidate reference genes. Since the expression of *18S* was exceptionally high and variable across the different treatments (Figure 1 and Table 2), it was excluded from further analyses.

### geNorm analysis

Next, the geNorm software was used to determine the expression stability of the selected candidate genes in different samples. The expression ratio of two suitable reference genes should be constant across different samples, which is the underlying principle followed by the geNorm program. Two parameters defined by the program were used to assess the stability of reference genes: M (average expression stability) and V (pairwise variation). In each group of samples, the M stability value for each gene, which is inversely related to expression stability, was obtained as the average pair-wise variation in the transcript levels of one gene with respect to all other reference genes tested. V values were determined with all other control genes as the SD of the logarithmically transformed expression ratios.

**Figure 2:**
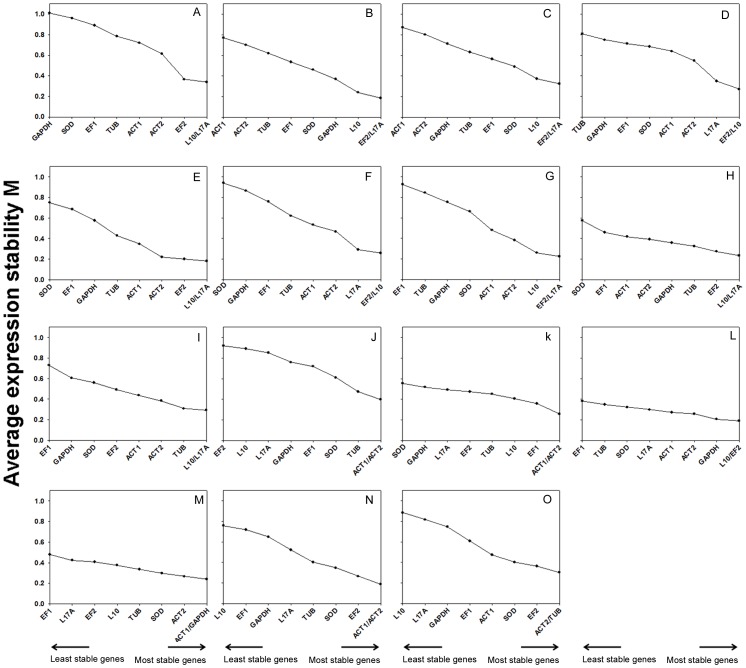
Average expression stability values (M) of the candidate reference genes for tissue samples. Average expression stability values (M) of the reference genes were measured during stepwise exclusion of the least stable reference genes. A lower M value indicates more stable expression, as analyzed by the geNorm software in *S.exigua* samples at five tissue samples in molting stage(B), five tissue samples in feeding stage(C), five tissue samples in wandering stage(D), epidermis samples in three specific larval physiological stages (E), fat body samples in three specific larval physiological stages (F), head samples in different stages (G), hemocytes samples in different stages (H), midgut samples in different stages (I), larvae samples (K), pupae samples (L), adult samples (M), male samples (N),female samples (O).The M values calculated for all the samples examined in all specific larval physiological stages(A) and all body samples examined in all developmental stages (J)are also given.

The gene with the highest M value was considered to have the least stable expression. Thus, the tested reference genes were ranked according to the stability of their expression by stepwise exclusion of the gene with the highest M value (Figure 2). Starting from the two most stable genes on the right, the genes are ranked according to reducing expression stability, ending with the least stable gene on the left. From all of the expression data of the tissue sample groups examined, *EF2*, *L10*, and *L17A* were the three most stable genes, suggesting that they play housekeeping roles and may be widely used for multiple conditions (Figure 2). While the *ACT1* and *ACT2* genes with an M value of 0.3990 were most stably expressed throughout the developmental stages (Figure 2J), *EF2* and *L10* showed the higher stable expression in pupae (Figure 2L). The highest ranked genes were *ACT1* and *ACT2* for larvae and male groups (Figure 2K, 2N); *ACT1* and *GAPDH* for adults (Figure 2M); and *ACT2* and *TUB* for female groups (Figure 2O). All tested reference genes reached high expression stability with M values below 1.1, far below the default limit of 1.5 for defining stably expressed genes.

**Figure 3:**
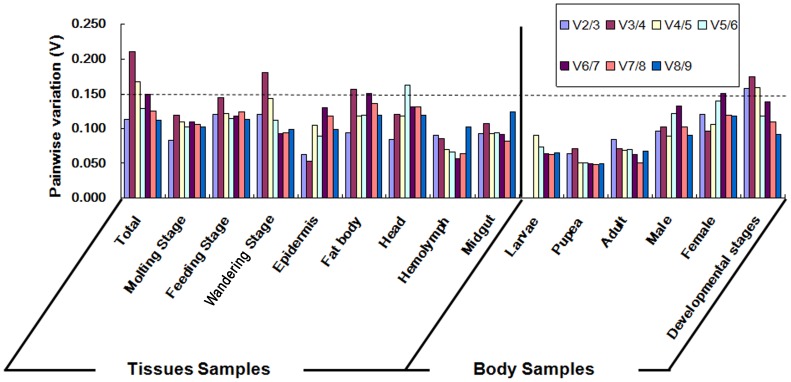
variation (V) analysis of the candidate reference genes. The pairwise variation (Vn/Vn+1) was analyzed between the normalization factors NFn and NFn+1 by the geNorm software to determine the optimal number of reference genes required for qRT-PCR data normalization.

It has been reported that more than one reference gene is required for accurate normalization [16]. When the use of additional genes was equally informative, the pairwise variation (Vn/Vn+1) between the sequential normalization factors (NF) (NFn and NFn+1) was calculated by geNorm to determine the optimal number of reference genes for each experimental condition (Figure 3). The cut-off value of pairwise variation of 0.15 was proposed to indicate that inclusion of an additional reference gene would be unnecessary.

Analysis of the pairwise variation in all developmental stages samples revealed a significant decrease with the inclusion of a fifth gene (Figure 3). Normalization factors should preferably consist of at least five reference genes, because the pairwise variation of the V2/3, V3/4, and V4/5 values were 0.158, 0.175, and 0.159, respectively, all of which exceeded the threshold of 0.15, while the pairwise variation of the V5/6 value was 0.1118. Based on this analysis, *EF1*, *SOD*, *TUB*, *ACT1* and *ACT2* should be ideal reference genes for normalizing gene expression data in all developmental stages samples. Analysis of the pairwise variation in other samples revealed that two reference genes may be sufficient to normalize expression values of target genes. Therefore, the two most stably expressed genes, mentioned above for each type of samples were selected as reference genes.

### NormFinder analysis

Finally, the NormFinder software tool was also employed to investigate each type of sample. This algorithm is used for identifying the optimal normalization gene among a set of candidate genes. When analyzing expression data using the qRT-PCR method, the software provides a stability value for each gene, which is the estimated expression variation if such gene is used for normalization. The candidate normalization genes were ranked according to the stability of their expression patterns between subgroups of the sample set under a given experimental condition. The lower average expression stability values represented more stable gene expression within the gene set examined.

Similarly to geNorm, the top-ranked candidates in different sample groups were analyzed by NormFinder (Table 3). Among all tissues, *L10* ranked one of the three most stable genes, while *EF2* and *L17A* ranked between the top four genes (except in hemolymph and midgut samples), in agreement with results of the other two programs. The ranking in the two sex sample groups showed significant differences compared with other body samples. For example, the *L10* gene ranked among the four most stable genes for pupae, adult and all larvae sample groups, but it ranked last in the sex sample groups. The *SOD* gene also ranked better in male and female samples than in others samples. All ranking results are summarized in Table S1.

**Table 3:** *S.exigua* reference genes for normalization and their expression stability values calculated by the NormFinder software.

Rank	Different tissues		Developmental life stages b	
	Total a	Epidermis	Fat body	Head	Hemolymph	Midgut	
	Gene	Stability	Gene	Stability	Gene	Stability	Gene	Stability	Gene	Stability	Gene	Stability	Gene	Stability
1	L10	0.223	ACT2	0.026	EF2	0.089	EF2	0.09	EF2	0.141	L17A	0.114	SOD	0.327
2	L17A	0.314	L17A	0.149	L10	0.089	ACT2	0.163	ACT2	0.184	ACT2	0.137	ACT2	0.339
3	EF2	0.457	L10	0.163	L17A	0.13	L10	0.209	L10	0.189	L10	0.2	GAPDH	0.393
4	ACT2	0.672	EF2	0.23	ACT2	0.438	L17A	0.244	ACT1	0.207	TUB	0.213	EF1	0.414
5	ACT1	0.88	ACT1	0.429	ACT1	0.489	ACT1	0.399	GAPDH	0.222	ACT1	0.265	TUB	0.471
6	SOD	0.889	GAPDH	0.456	EF1	0.639	SOD	0.582	L17A	0.227	SOD	0.421	ACT1	0.478
7	TUB	0.91	TUB	0.538	GAPDH	0.643	GAPDH	0.661	EF1	0.306	EF2	0.454	EF2	0.552
8	EF1	0.917	EF1	0.581	TUB	0.652	EF1	0.731	TUB	0.318	GAPDH	0.458	L17A	0.558
9	GAPDH	0.981	SOD	0.601	SOD	0.732	TUB	0.764	SOD	0.639	EF1	0.763	L10	0.586

Rank	Specific Larval Physiological Stages			Developmental life stages		Sex
	Molting Stage	Feeding Stage	Wandering Stage		Larvae	Pupae	Adult	Male	Female
	Gene	Stability	Gene	Stability	Gene	Stability	Gene	Stability	Gene	Stability	Gene	Stability	Gene	Stability	Gene	Stability
1	L10	0.178	L10	0.007	L10	0.341	L10	0.121	ACT2	0.095	ACT2	0.084	SOD	0.208	EF2	0.182
2	L17A	0.284	L17A	0.238	L17A	0.374	EF1	0.224	ACT1	0.122	GAPDH	0.123	L17A	0.246	EF1	0.282
3	EF2	0.329	EF2	0.372	ACT2	0.452	ACT1	0.23	GAPDH	0.151	ACT1	0.21	TUB	0.258	SOD	0.322
4	SOD	0.477	EF1	0.469	EF2	0.503	ACT2	0.274	L10	0.154	L10	0.217	ACT2	0.348	TUB	0.375
5	GAPDH	0.582	TUB	0.569	ACT1	0.603	L17A	0.283	EF2	0.183	SOD	0.234	ACT1	0.4	ACT2	0.411
6	TUB	0.661	SOD	0.717	EF1	0.612	EF2	0.29	SOD	0.193	EF2	0.261	GAPDH	0.477	GAPDH	0.499
7	ACT2	0.694	ACT2	0.826	GAPDH	0.693	TUB	0.299	L17A	0.205	TUB	0.265	EF2	0.495	L17A	0.544
8	EF1	0.702	GAPDH	0.982	SOD	0.733	GAPDH	0.313	TUB	0.265	L17A	0.28	EF1	0.501	ACT1	0.695
9	ACT1	0.913	ACT1	1.005	TUB	0.871	SOD	0.401	EF1	0.299	EF1	0.414	L10	0.554	L10	0.727

^a^ Total, all the tissue samples in three specific physiological stages;

^b^ Developmental Stages, all the developmental life stages samples.

### Consensus list of reference genes

Because of the different algorithms used and the different sensitivities toward co-regulated reference gene candidates, the three software tools offered different ranks in each sample group. Although rankings of the most suitable reference genes were not identical, the three best reference genes identified by the different methods were similar, and they only varied in their relative rank positions (Table S1). Finally, the highest ranking reference genes were identified (Table 4). Interestingly, *L10, EF2* and *L17A* ranked highest in different tissue groups, and *ACT2* ranked as the fourth most stable (except in molting stage samples), indicating that these four genes could be selected as the best reference genes for tissue research in *S. exigua*.

**Table 4:** The best-ranking reference genes across different experimental conditions in *S. exigua* according to software analysis.

Experimental conditions		The best-ranking reference genes		
	**Molting Stage**	*EF2*	*L10*	*L17A*
**Specific Larval Physiological Stages**	**Feeding Stage**	*L17A*	*L10*	*EF2*
	**Wandering Stage**	*L17A*	*L10*	
	**Total** a	*L10*	*L17A*	*EF2*
	**Epidermis**	*ACT2*	*L17A*	*L10*
	**Fat body**	*EF2*	*L10*	*L17A*
**Different Tissues**	**Head**	*EF2*	*L17A*	*L10*
	**Hemolymph**	*EF2*	*L10*	*L17A*
	**Midgut**	*L17A*	*L10*	
**Developmental Stages** b		*SOD*	*ACT2*	*GAPDH*
	**Larvae**	*ACT1*	*ACT2*	*L10*
**Developmental life stages**	**Pupae**	*GAPDH*	*ACT2*	*ACT1*
	**Adult**	*GAPDH*	*ACT2*	*ACT1*
**Sex**	**Male**	*SOD*	*L17A*	
	**Female**	*EF2*	*SOD*	

^a^ Total, all the tissues samples in three Specific Larval Physiological Stages;

^b^ Developmental Stages samples, all the developmental life stages samples.

In different whole body samples, the last ranking reference genes showed significant differences. The best reference genes were *ACT2, ACT1* and *L10* for larvae groups; *GAPDH, ACT1* and *ACT2* for pupae and adults; *SOD* and *L17A* for males; and *EF2* and *SOD* for females (Table 4). Thus, for all developmental stage samples, using the five genes *SOD, ACT2, GAPDH, EF1* and ACT1 together should provide reliable results in expression studies of *S. exigua*.

### Target gene expression

In order to demonstrate the effect of reference genes on target gene expression data, the relative expression of the target gene *DSP* was investigated under different experimental conditions. Target expression analyses further showed that differences in quantification were detected when normalizing with arbitrary internal controls relative to the best reference genes. The best or the most unstable reference genes were selected based on their rank order of expression stability among the 10 candidates evaluated in this study.

**Figure 4:**
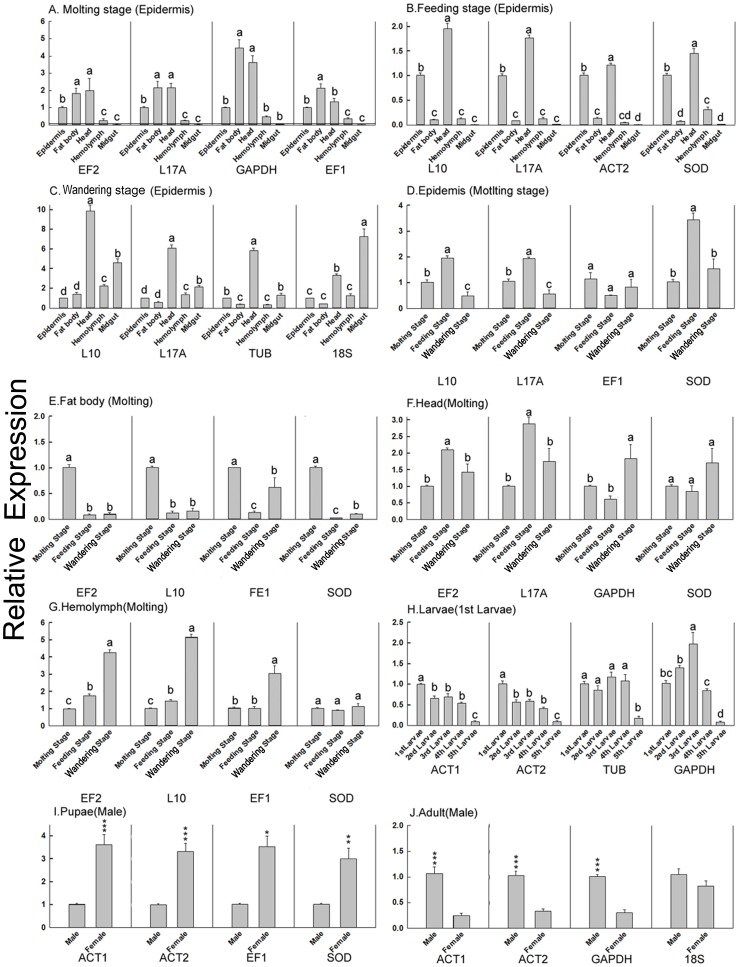
Analysis of expression of the target gene *DSP* using different reference genes. The relative expression of the target gene *DSP* among different samples normalized with different reference genes was investigated. Control groups used in each sample set were: A. molting stage (epidermis): epidermis samples in molting stage; B. feeding stage (epidermis): epidermis samples in feeding stage; C. wandering stage (epidermis): epidermis samples in wandering stage; D. epidermis (molting stage): epidermis samples in molting stage; E. fat body (molting stage): fat body samples in molting stage; F. head (molting stage): head samples in molting stage; G. hemolymph (molting stage): hemolymph samples in molting stage; H. larvae (1^st^ larvae): 1^st^ larvae samples; I. pupae (male): the male pupae samples; J. adult (male): male adult samples. Data are means ± SEM. The comparisons among more than two reference genes were analyzed using one-way ANOVA (from A to H). Those between two reference genes were compared using Student's t-test (I & J). *P<0.05; **P<0.01; ***P<0.001.

Arbitrary selection of reference genes may thus decrease the accuracy of calculating target gene expression, since such a normalization strategy can over-estimate or under-estimate differences in expression level among different samples. For example, the relative expression level of *DSP* showed no significant differences between adult male and female samples when calculated using 18S as the reference gene; however, its expression was significantly different when normalized by other reference genes (such as *ACT1*, *ACT2*) (Figure 4J). Similar changes also occurred in calculating relative expression levels of *DSP* after normalization by other unstable reference genes, such as *GAPDH* in the molting stage, *TUB* in the molting stage, *SOD* and *18S* in the epidermis, *SOD* in the head, *EF1* and *SOD* in hemolymph, *TUB* in all larvae groups and *EF1* and *SOD* in pupae (Figure 4A, 2C, 2D, 2F, 2G, 2H and 2I).

Relative expression levels of the target gene in different samples were even more divergent if calculated using arbitrary reference genes. For example, when using *EF1* or *SOD* as reference gene, the fat body *DSP* expression in the feeding stage was lower than that in the wandering stage; however, after using other reference genes, the conclusion was modified, and they were determined to be at the same level (Figure 4E). Similar errors could be produced when using *EF1* in the molting stage, and *ACT2* or *SOD* in the feeding stage (Figure 4A, B). Thus, determination of the optimal reference genes is important for accurate normalization of qRT-PCR data, especially when differences in expression levels are subtle.

Inaccurate conclusions were made when certain reference genes were used for normalization. While the larvae *DSP* expression level was higher in 3^rd^ larvae when using *GAPDH* as the reference gene, it exhibited a significant age-dependent decrease in larvae when normalizing with *ACT1* or *ACT2* (Figure 4H). Similar results were found when using *18S* in the molting stage and *GAPDH* in the head tissue (Figure4C, 4F). Taken together, results of this study showed that the selection of reference genes for qRT-PCR data normalization varied on a case-by-case basis. Thus, in order to obtain accurate expression data, any given sample set must be assessed using the panel of selected candidate reference genes.

## Discussion

qRT-PCR quantification requires robust normalization by reference genes to offset confounding variations in experimental data. However, improper selection of reference genes can conceal or magnify real biological changes due to changes in the reference gene expression [50], [51]. Furthermore, using a single endogenous control can also profoundly influence the statistical outcome and may lead to inaccurate data interpretation [52]. Each candidate reference gene should be evaluated under specific experimental conditions for gene profiling to ensure a constant level of expression [40]. In this study, we examined 10 candidate reference genes from *S. exigua* and analyzed the stability of these genes across various sample sets using three analytical software programs. These genes involved in ubiquitous cellular processes represent those commonly used as single normalizer in *S. exigua* gene expression studies. The studied sample subgroups included detailed life developmental stages (prepupae and every instar of the larvae), both sexes and five different tissues in three larval physiological stages. Although different ranks were offered by the three analytical tools, the combined results ultimately provided recommendations for the optimal reference genes. The different rankings of the reference genes in different sample sets in this study illustrated the need for evaluating their use under various experimental conditions. Compared with a previous report [39], our work provides a more complete set of information for the selection of reference genes in *S. exigua*.

A major conclusion arising from our results is that none of the candidate reference genes could serve as a “universal” normalizer that would maintain a constant expression level across all experimental conditions. Although most of these candidates are considered to be classical housekeeping genes and are widely used for data normalization, they exhibited considerable variations in expression stability among the different samples. The results of this study emphasized that the stability of reference gene expression must be verified under all experimental conditions to be investigated. Given that all internal reference genes are regulated to some extent and if none are constitutively expressed for each experimental treatment, then a combination of reference genes that would best fulfill the universality criteria should be selected (i.e., *L10, EF2*, and *L17A* across different tissue subgroups in our study).

In contrast to the findings of Teng *et al* [39], who selected relatively stable reference genes just from four candidate housekeeping genes in certain tissues from final instar larvae and developmental stages, we investigated the influence of many more variables (e.g. five different tissues in three larval physiological stages and life developmental stages) on the expression stability of the studied genes. The authors of the study above chose *GAPDH* as one of the most stable reference genes, while *GAPDH* ranked last among the reference genes across most sample sets in the current study (Table S1). Assessing a low number of initial candidate genes would lead to such a misleading result. Although the *GAPDH* gene ranked in the first three in all developmental stages sample subgroup, normalizing the expression of a target gene just by one reference gene is not ideal. Since the *GAPDH* gene ranked highly in some sample subgroups, it may be used as the reference gene in certain experimental conditions but not as a sole universal normalizer.

While stability of reference genes still must be determined on a case-by-case basis in *S. exigua* studies, certain genes may be preferred for normalization in experiments involving different treatments. The lowest ranking reference genes also showed significant differences across different whole body sample subgroups. This observation indicated that candidate genes in body subgroups showed more variations than in tissue subgroups.

Results of this study suggest that more complex sample sets will exhibit higher variability in the reference genes. To determine the optimal number of reference genes, the pairwise variation (Vn/Vn+1) between the sequential NF (NFn and NFn+1) was calculated by geNorm. After the analysis, two reference genes were found to be sufficient for normalizing expression values of target genes in most of the samples, but five reference genes were needed in all of the life developmental stages samples (Figure 3), indicating that larger sample sizes require a higher number of reference genes for accurate normalization. The same results were obtained in a *Drosophila* reference gene selection study [53]. Along the same way, in an aging-related study, nine reference genes were sufficient for three samples, whereas 13 reference genes were needed when nine samples were tested. Finally in a neurodegeneration-related study, one reference gene was feasible for three samples; however, the number of the reference genes needed to be increased to six for accurate normalization in nine samples. Perhaps additional reference genes are required when adding more samples into a study, because it would be harder to reach the minimum value of Vn/n+1, due to the introduction of more unstable factors.

Compared to the other reference genes tested, *18S* ranked low in most experimental conditions and displayed an excessively high expression level, excluding it as a potential reference gene. However, the differences in rRNA and mRNA fractions between samples limit the use of *18S* as a normalizer in qRT-PCR analyses [1], [40], [54]. In other words, rRNA cannot be used for correcting sample-to-sample variation in the quantity of mRNA, as it has been shown on occasion to fail to be representative of mRNA levels [55]. This shortcoming may explain the high coefficient of variation of *18S* in our study. The low Ct values of *18S* observed in our study (Figure 1) reflect the abundance of these transcripts. Thus, in order to use *18S* as a reference gene, the samples would need to be diluted sufficiently to keep the rRNA within the range of detection. However, the target gene would also be diluted further, potentially leading to an over-estimation or under-estimation in differences of target gene expression among different conditions (Figure 4). Therefore, we suggest that*18S* should be excluded as a reference gene in qRT-PCR, since the expression of other candidates proved to be more stable.

*SOD* was never listed in the top three ranked reference genes across tissue sample sets and some of the whole body sample sets in our study (Table 4 & Table S1). Similarly, the glycolytic enzyme *GAPDH* was selected as a suitable reference gene for only three out of fifteen samples (i.e., pupae and adult), even though it has been reported as a good normalizer in previous gene expression studies of *S. exigua* and other insect species [38]. In contrast, *L10* and *L17A*, were found to be stably expressed across the different samples. Though they all encode the structural constituents of the ribosome, it was reported that *L17A* was related to the pupal diapause regulation in the insect [56] and *L10* was a component of an antiviral signaling [57]. These observations indicate that the two ribosomal protein genes are not co-regulated and can be regarded as independent reference genes. Additionally, *EF2* ranked at the top as a reference gene in most samples in this study, but it has rarely been previously used as a normalizer. Interestingly, *EF1* ranked last in some sample sets in our study. The elongation factors (i.e., *EF1* & *EF2*) play an important role in translation by catalyzing GTP-dependent binding of aminoacyl tRNA to the acceptor site of the ribosome. Recently, a number of studies have reported that it is a suitable reference gene in different species, including salmon [58], [59], humans [60] and Orthoptera [61], [62].

Actin, as the major component of the protein scaffold which supports the cell and determines its shape, was also selected as a good reference gene under some conditions. It was expressed at moderately abundant levels in most samples. Even though two actin genes were selected as candidate reference genes in our study, they have been reported to be unsuitable for normalizing qRT-PCR data due to large measurement errors [4]. On the other hand, actin has ranked at the top as a reference gene in expression studies in the desert locust [61], European honey bee [38], two species of Collembola [63] and the salmon louse [58].

Taken together, the simultaneous measurement of a panel of candidate reference genes is essential for quantification by qRT-PCR. As empirically-determined or pre-validated reference genes may yield inaccurate results, data normalization needs to be optimized for each particular assay.

## Conclusion

In our study, several reference genes suitable for normalizing qRT-PCR data in *S. exigua* were identified. Although most of the selected candidates exhibited stable expression patterns acceptable for reference genes, some showed the highest stability in different experimental conditions. While the expression levels of *L10, EF2*, and *L17A* were most stable in different tissue sample sets, the best reference genes selected were *ACT2, ACT1*, and *L10* for larvae samples; *GAPDH, ACT1*, and *ACT2* for pupae and adults; *SOD* and *L17A* for males; and *EF2* and *SOD* for females. Overall, five genes, *SOD, ACT2, GAPDH, EF1*, and *ACT1*, were determined to be most reliable when used together to analyze all developmental stage sample groups in *S. exigua*.

## Supporting Information

Table S1 **Ranking of candidate reference genes according to their stability value using BestKeeper, geNorm, and NormFinder analyses.** Candidates are listed from top to bottom in order of decreasing expression stability.
(DOC)
