# Supplementary material for: Selection and Evaluation of Reference Genes for Expression Analysis Using qRT-PCR in the Beet Armyworm Spodoptera exigua (Hübner) (Lepidoptera: Noctuidae)
Source: PLoS One. 2014 Jan 15;9(1):e84730. doi: 10.1371/journal.pone.0084730 (PMC3893131; doi:10.1371/journal.pone.0084730)
Supplement: Table S1 — Ranking of candidate reference genes according to their stability value using BestKeeper, geNorm, and NormFinder analyses. Candidates are listed from top to bottom in order of decreasing expression stability. (DOC) [file pone.0084730.s001.doc]

**Supplementary materials**

**Table.S1 Ranking of candidate reference genes according to their stability value using BestKeeper, geNorm, and NormFinder analyses. Candidates are listed from top to bottom in order of decreasing expression stability**

|  |  |  |  |  |  |  |  | **Different** | **tissues** |  |  |  |  |  |  |  |
| --- | --- | --- | --- | --- | --- | --- | --- | --- | --- | --- | --- | --- | --- | --- | --- | --- |
| **Rank** |  |  |  | **Epidermis** |  |  |  | **Fat body** |  |  |  | **Head** |  |  |  | **Hemolymph** |
|  | **Bestkeeper** | **geNorm** | **NormFinder** | **consensus** | **Bestkeeper** | **geNorm** | **NormFinder** | **consensus** | **Bestkeeper** | **geNorm** | **NormFinder** | **consensus** | **Bestkeeper** | **geNorm** | **NormFinder** | **consensus** |
| 1 | *SOD* | *L17A* | *ACT2* | *ACT2* | *L10* | *EF2* | *EF2* | *EF2* | *ACT2* | *EF2* | *EF2* | *EF2* | *EF2* | *L17A* | *EF2* | *EF2* |
| 2 | *GAPDH* | *L10* | *L17A* | *L17A* | *EF2* | *L10* | *L10* | *L10* | *EF2* | *L17A* | *ACT2* | *L17A* | *L10* | *L10* | *ACT2* | *L10* |
| 3 | *ACT2* | *EF2* | *L10* | *L10* | *L17A* | *L17A* | *L17A* | *L17A* | *ACT1* | *L10* | *L10* | *L10* | *L17A* | *EF2* | *L10* | *L17A* |
| 4 | *EF1* | *ACT2* | *EF2* | *GAPDH* | *GAPDH* | *ACT2* | *ACT2* | *ACT2* | *L10* | *ACT2* | *L17A* | *ACT2* | *ACT2* | *TUB* | *ACT1* | *ACT2* |
| 5 | *ACT1* | *ACT1* | *ACT1* | *ACT1* | *EF1* | *ACT1* | *ACT1* | *ACT1* | *L17A* | *ACT1* | *ACT1* | *ACT1* | *ACT1* | *GAPDH* | *GAPDH* | *ACT1* |
| 6 | *L17A* | *TUB* | *GAPDH* | *EF2* | *ACT2* | *TUB* | *EF1* | *EF1* | *TUB* | *SOD* | *SOD* | *SOD* | *GAPDH* | *ACT2* | *L17A* | *GAPDH* |
| 7 | *L10* | *GAPDH* | *TUB* | *SOD* | *ACT1* | *SEF1* | *GAPDH* | *GAPDH* | *EF1* | *GAPDH* | *GAPDH* | *GAPDH* | *EF1* | *ACT1* | *EF1* | *TUB* |
| 8 | *EF2* | *EF1* | *EF1* | *EF1* | *TUB* | *GAPDH* | *TUB* | *TUB* | *SOD* | *TUB* | *EF1* | *EF1* | *TUB* | *EF1* | *TUB* | *EF1* |
| 9 | *TUB* | *SOD* | *SOD* | *TUB* | *SOD* | *SOD* | *SOD* | *SOD* | *GAPDH* | *EF1* | *TUB* | *TUB* | *SOD* | *SOD* | *SOD* | *SOD* |
|  |  |  |  |  | **Different** | **tissues** |  |  |  |  |  | **Sex** |  |  |  |  |
| **Rank** |  |  |  | **Midgut** |  |  |  | **Total ^a^** |  |  |  | **Male** |  |  |  | **Female** |
|  | **Bestkeeper** | **geNorm** | **NormFinder** | **consensus** | **Bestkeeper** | **geNorm** | **NormFinder** | **consensus** | **Bestkeeper** | **geNorm** | **NormFinder** | **consensus** | **Bestkeeper** | **geNorm** | **NormFinder** | **consensus** |
| 1 | *L10* | *L17A* | *L17A* | *L17A* | *L10* | *L10* | *L10* | *L10* | *L17A* | *ACT1* | *SOD* | *SOD* | *SOD* | *ACT2* | *EF2* | *EF2* |
| 2 | *L17A* | *L10* | *ACT2* | *L10* | *L17A* | *L17A* | *L17A* | *L17A* | *TUB* | *ACT2* | *L17A* | *L17A* | *EF2* | *TUB* | *EF1* | *SOD* |
| 3 | *TUB* | *TUB* | *L10* | *TUB* | *EF2* | *EF2* | *EF2* | *EF2* | *SOD* | *EF2* | *TUB* | *TUB* | *EF1* | *EF2* | *SOD* | *EF1* |
| 4 | *EF2* | *ACT2* | *TUB* | *ACT2* | *ACT2* | *ACT2* | *ACT2* | *ACT2* | *GAPDH* | *SOD* | *ACT2* | *ACT2* | *GAPDH* | *SOD* | *TUB* | *TUB* |
| 5 | *SOD* | *ACT1* | *ACT1* | *EF2* | *ACT1* | *ACT1* | *ACT1* | *ACT1* | *L10* | *TUB* | *ACT1* | *ACT1* | *L17A* | *ACT1* | *ACT2* | *GAPDH* |
| 6 | *GAPDH* | *EF2* | *SOD* | *SOD* | *TUB* | *TUB* | *SOD* | *TUB* | *EF1* | *L17A* | *GAPDH* | *GAPDH* | *L10* | *EF1* | *GAPDH* | *ACT2* |
| 7 | *ACT2* | *SOD* | *EF2* | *ACT1* | *SOD* | *EF1* | *TUB* | *SOD* | *ACT2* | *GAPDH* | *EF2* | *EF2* | *TUB* | *GAPDH* | *L17A* | *L17A* |
| 8 | *ACT1* | *GAPDH* | *GAPDH* | *GAPDH* | *EF1* | *SOD* | *EF1* | *EF1* | *ACT1* | *EF1* | *EF1* | *EF1* | *ACT2* | *L17A* | *ACT1* | *ACT1* |
| 9 | *EF1* | *EF1* | *EF1* | *EF1* | *GAPDH* | *GAPDH* | *GAPDH* | *GAPDH* | *EF2* | *L10* | *L10* | *L10* | *ACT1* | *L10* | *L10* | *L10* |
|  |  |  |  | **Special** | **Larval** | **Physiological** | **Stages** |  |  |  |  |  | **Developmental** | **life** | **stages ^b^** |  |
| **Rank** |  |  | **Molting** | **Stage** |  |  |  | **Feeding Stage** |  |  | **Wandering** | **Stage** |  |  |  |  |
|  | **Bestkeeper** | **geNorm** | **NormFinder** | **consensus** | **Bestkeeper** | **geNorm** | **NormFinder** | **consensus** | **Bestkeeper** | **geNorm** | **NormFinder** | **consensus** | **Bestkeeper** | **geNorm** | **NormFinder** | **consensus** |
| 1 | *L10* | *L10* | *EF2* | *EF2* | *L10* | *L10* | *L17A* | *L17A* | *L10* | *L10* | *L10* | *L17A* | *SOD* | *ACT2* | *SOD* | *SOD* |
| 2 | *L17A* | *L17A* | *L17A* | *L10* | *L17A* | *L17A* | *EF2* | *L10* | *L17A* | *L17A* | *EF2* | *L10* | *EF1* | *ACT1* | *ACT2* | *ACT2* |
| 3 | *EF2* | *EF2* | *L10* | *L17A* | *EF2* | *EF2* | *L10* | *EF2* | *EF1* | *ACT2* | *L17A* | *ACT1* | *L17A* | *TUB* | *GAPDH* | *GAPDH* |
| 4 | *SOD* | *SOD* | *GAPDH* | *TUB* | *TUB* | *EF1* | *SOD* | *TUB* | *EF2* | *EF2* | *ACT2* | *ACT2* | *GAPDH* | *SOD* | *EF1* | *EF1* |
| 5 | *GAPDH* | *GAPDH* | *SOD* | *SOD* | *ACT2* | *TUB* | *EF1* | *EF1* | *GAPDH* | *ACT1* | *ACT1* | *EF2* | *L10* | *EF1* | *TUB* | *ACT1* |
| 6 | *ACT2* | *TUB* | *EF1* | *GAPDH* | *ACT1* | *SOD* | *TUB* | *SOD* | *ACT1* | *EF1* | *SOD* | *GAPDH* | *ACT2* | *GAPDH* | *ACT1* | *TUB* |
| 7 | *ACT1* | *ACT2* | *TUB* | *ACT2* | *EF1* | *ACT2* | *GAPDH* | *ACT1* | *ACT2* | *GAPDH* | *EF1* | *SOD* | *ACT1* | *L17A* | *EF2* | *L17A* |
| 8 | *TUB* | *EF1* | *ACT2* | *EF1* | *SOD* | *GAPDH* | *ACT2* | *ACT2* | *SOD* | *SOD* | *GAPDH* | *EF1* | *TUB* | *L10* | *L17A* | *L10* |
| 9 | *EF1* | *ACT1* | *ACT1* | *ACT1* | *GAPDH* | *ACT1* | *ACT1* | *GAPDH* | *TUB* | *TUB* | *TUB* | *TUB* | *EF2* | *EF2* | *L10* | *EF2* |
|  |  |  |  |  |  |  |  |  | **Developmental life stages** |  |  |  |  |  |  |  |
|  |  | **Rank** |  |  |  | **Larvae** |  |  |  | **Pupae** |  |  |  | **Adult** |  |  |
|  |  |  | **Bestkeeper** | **geNorm** | **NormFinder** | **consensus** | **Bestkeeper** | **geNorm** | **NormFinder** | **consensus** | **Bestkeeper** | **geNorm** | **NormFinder** | **consensus** |  |  |
|  |  | 1 | *L10* | *ACT1* | *L10* | *ACT1* | *SOD* | *EF2* | *ACT2* | *GAPDH* | *ACT2* | *ACT1* | *ACT2* | *GAPDH* |  |  |
|  |  | 2 | *EF2* | *ACT2* | *EF1* | *ACT2* | *ACT2* | *L10* | *ACT1* | *ACT2* | *ACT1* | *GAPDH* | *GAPDH* | *ACT2* |  |  |
|  |  | 3 | *GAPDH* | *EF1* | *ACT1* | *L10* | *ACT1* | *GAPDH* | *GAPDH* | *ACT1* | *SOD* | *ACT2* | *ACT1* | *ACT1* |  |  |
|  |  | 4 | *L17A* | *L10* | *ACT2* | *EF1* | *TUB* | *ACT2* | *L10* | *L10* | *TUB* | *SOD* | *L10* | *SOD* |  |  |
|  |  | 5 | *EF1* | *TUB* | *L17A* | *L17A* | *GAPDH* | *ACT1* | *EF2* | *EF2* | *GAPDH* | *TUB* | *SOD* | *TUB* |  |  |
|  |  | 6 | *TUB* | *EF2* | *EF2* | *TUB* | *L10* | *L17A* | *SOD* | *SOD* | *EF2* | *L10* | *EF2* | *L10* |  |  |
|  |  | 7 | *ACT1* | *L17A* | *TUB* | *EF2* | *L17A* | *SOD* | *L17A* | *L17A* | *L17A* | *EF2* | *TUB* | *EF2* |  |  |
|  |  | 8 | *SOD* | *GAPDH* | *GAPDH* | *SOD* | *EF2* | *TUB* | *TUB* | *TUB* | *L10* | *L17A* | *L17A* | *L17A* |  |  |
|  |  | 9 | *ACT2* | *SOD* | *SOD* | *GAPDH* | *EF1* | *EF1* | *EF1* | *EF1* | *EF1* | *EF1* | *EF1* | *EF1* |  |  |

**^a^** Total, all the tissues samples in three special larval physiological stages; **^b^** Developmental Stages samples, all the developmental life stages samples.
